# Rhizosphere-associated bacterial and fungal communities of two maize hybrids under increased nitrogen fertilization

**DOI:** 10.3389/fpls.2025.1549995

**Published:** 2025-03-03

**Authors:** Qing Liu, HongCui Dai, Hao Cheng, Guodong Shao, Liang Wang, Hui Zhang, Yingbo Gao, Kaichang Liu, Xiaomei Xie, Junhua Gong, Xin Qian, Zongxin Li

**Affiliations:** ^1^ State Key Laboratory of Nutrient Use and Management, Shandong Academy of Agricultural Sciences, Jinan, China; ^2^ Geo-Biosphere Interactions, Department of Geosciences, University of Tuebingen, Tuebingen, Germany; ^3^ Engineering Technology Research Institute, Shikefeng Chemical Industry Co., Ltd, Linyi, China

**Keywords:** maize hybrids, nitrogen rates, high-throughput sequencing, community assembly, microbial function

## Abstract

**Introduction:**

The selection and application of nitrogen-efficient maize hybrids have significantly bolstered contemporary food security. Nevertheless, the effects of heightened nitrogen fertilizer demand of these crops on the composition and assembly of soil microbial communities in agricultural production require further elucidation.

**Methods:**

In this study, the effects of four nitrogen fertilizer managements on rhizosphere bacterial and fungal community assembly, co-occurrence network and function of two maize hybrids (LD981 and DH605) were compared.

**Results and discussion:**

Findings revealed that the bacterial community was primarily shaped by deterministic processes, while stochastic processes played a pivotal role in fungal community assembly. N-efficient hybrid DH605 had a more stable microbial network than N-inefficient hybrid LD981. At N3 (130 g N/m^2^) rate, the bacterial and fungal community networks were the most complex but unstable, followed by N2 (87 g N/m^2^), N0 (0 g N/m^2^), and N1 (43 g N/m^2^) rates. Excessive nitrogen rate (N3) increased the relative abundance of denitrification genes *nirK* and *norB* by enriching nitrogen-related genus such as *Nitrolancea* and *Nitrosospira*. It led to an increase in the relative abundance of pathways such as cysteine and methionine metabolism and pyruvate metabolism. The effects of management practices (i.e. maize hybrids and N rates) on microbial communities were ultimately directly or indirectly reflected in microbial functions. Our findings illustrate the relationship between the appropriate selection of crop hybrids and management measures in optimizing rhizosphere microbial community assembly and promoting nitrogen use, which is necessary for sustainable food security.

## 1 Introduction

Nitrogen (N) fertilization is crucial for enhancing crop yield and ensuring food security (Snapp et al., 2023). Globally, the application of N fertilizer has increased considerably and is expected to increase almost threefold (260 Tg N yr^–1^) by 2050 (Mogollon et al., 2018) to satisfy the escalating demand for agricultural products driven by population growth (Mueller et al., 2012). Nitrogen exceeding the planetary boundaries makes the great demand to increase nitrogen use efficiency (Schulte-Uebbing et al., 2022). In agroecosystems, an increase in crop productivity is usually associated with N fertilization-induced changes in soil nutrient availability as well as microbial communities and activities (Muhammad et al., 2022; Piazza et al., 2020). However, overuse of N fertilizer contributes to severe environmental risks at local and global scales, such as decreasing N use efficiency (Shao et al., 2023), accelerating soil acidification (Tkaczyk et al., 2020), stimulating nitrous oxide emissions (Pan et al., 2023), and degrading the quality of ground surface water (Hao et al., 2024). Thus, N fertilization management that optimizes crop production while simultaneously decreasing the impact on the environment is desirable.

Soil microorganisms regulate global biogeochemical cycles and are integral to maintaining soil ecological functions (Chen et al., 2017). They directly participate in the nutrient cycle, and energy transfer (Cui et al., 2019). The rhizosphere serves as a critical interface for interactions among plants, soil, and microbes, influenced by various environmental factors. Under different N fertilization rates, plant roots recruit specific root microorganisms from the soil, thus affecting crop yield in agroecosystems (Chen and Huang, 2021). Dong et al. (2021) reported that excessive N fertilizer usage adversely impacted bacterial communities responsible for nitrification and nitrogen fixation within the rhizosphere. Additionally, the abundance of plant pathogens was found to increase under high nitrogen fertilization conditions. Moreover, the fungal community in the rhizosphere also responded significantly to N fertilization rates. Paungfoo-Lonhienne et al. (2015) conducted a field experiment and reported that the N fertilization had a great effect on the rhizosphere fungal community composition instead of its richness. High N fertilization negatively affects carbon cycling in soil and enhances the growth of fungi with known pathogenic traits. Therefore, understanding how N fertilization regulates rhizosphere-associated microbiomes is of great agronomic interest.

The survival strategies of soil microorganisms, driven by community assembly, promote the mechanisms by which microbial structures are formed and are essential for ecosystem function (Anthony et al., 2020). Determinism, rooted in niche theory, highlights the impact of environmental filtration and biological interactions on microorganisms. Stochasticity, founded on neutral theory, emphasizes the role of random disturbances, birth-death events, and dispersal in shaping microbial communities (Zhu et al., 2024). The interplay between these assembly processes is modulated by environmental factors. For example, an increase in soil organic matter reduces the competition of microorganisms for limited resources, which leads to a shift from deterministic to stochastic process (Dini-Andreote et al., 2015). The addition of nitrogen significantly increases the significance of stochastic processes, independent of phylogenetic considerations (Zhou et al., 2022). Although communities dominated by stochasticity may encompass a greater variety of species capable of utilizing diverse resources, deterministic selection becomes crucial when microorganisms adapt their niches in response to resource scarcity (Li et al., 2023). For example, the N uptake of plants affects the ecological selection of soil microorganisms, so only those microorganisms that can tolerate nitrogen limitation (i.e., with high N mineralization potential) are able to survive (Zhang et al., 2024b). Thus, investigating the interactions between crops and microbes, as well as their implications for soil microbial assembly processes, may enhance our understanding of how various management practices affect soil functionality.

Maize (*Zea mays* L.) is a crucial staple food that plays various roles in global agri-food systems (Erenstein et al., 2022). Shao et al. (2023) found that N-efficient maize hybrids presented greater N use efficiency (NUE) than N-inefficient hybrids under low-N and excess-N conditions. However, there remains a gap in knowledge regarding the effects of N fertilization, maize hybrids, and their interactions on the microbial communities within the soil rhizosphere. To address this, we conducted a pot experiment under field conditions with two maize hybrids and four N fertilization rates and analyzed the soil physiochemical properties after the maize was harvested. The soil bacterial and fungal community compositions were examined via high-throughput sequencing to determine the responses of the soil microbial communities to N application and maize hybrid. The aims of this study were (1) to comprehensively understand shifts in soil rhizosphere microbial communities (community composition, assembly, co-occurrence network, and function) caused by N fertilization rates across N-efficient and N-inefficient maize hybrids and (2) to elucidate the correlations among the rhizosphere microbial community, physiochemical properties, and management practices (maize hybrids and N rates).

## 2 Materials and methods

### 2.1 Study site and experimental design

This study was carried out the Longshan Research Station (117°32 ′E, 36°43′ N) in Jinan City, Shandong Province, Eastern China. The site has a warm temperate monsoon climate, with a 30-year (1981–2010) mean annual precipitation of 693.4 mm and a mean annual temperature of 13.6°C. The studied soil was arable and was classified as Luvisols (IUSS Working Group WRB 2014). The general soil properties in the top 20 cm were as follows: pH, 7.31; soil organic carbon, 19.35 g kg^–1^; total N, 1.53 g kg^–1^; available N, 100.54 mg kg^–1^; available phosphorus, 57.01 mg kg^–1^; available potassium, 401.96 mg kg^–1^.

The field pot experiment was conducted using a split-plot design with two maize hybrids, LD981 and DH605, as the main plots, and four N fertilization rates in the form of urea at rates of 0, 6, 12, and 18 g N plant^–1^, equally to 0 (N0), 43 (N1), 87 (N2), and 130 (N3) g N m^-2^, with three replicates as the subplots. For each pot, 40% of the total N fertilizer was manually spread over the soil surface and integrated with the soil layer of 0–20 cm at the time of sowing, while 60% of the total N fertilizer was top-dressed at the physiological maturity phase. For all treatments, potassium (K) and phosphorus (P) fertilizers were used in the forms of potassium chloride (KCl, 5.5 g plant^–1^) and calcium dihydrogen phosphate (Ca(H_2_PO_4_)_2_, 16.4 g plant^–1^), respectively, and were applied once as basal fertilizers while sowing. A pottery pot with a height of 52.0 cm and a diameter of 42.0 cm was used in this experiment. The topsoil (0-20 cm) of winter wheat-summer maize rotation farmland was taken, and 42 kg of air-dried farmland soil were filled in each pot. The pottery pot was buried underground, and the pot edge was exposed to the surface for 5 cm. Three maize seeds were sown per pot in June 2019 and reduced to one seedling after four leaves emerged. Adequate water was provided during maize growth, and other managements were the same as high-yield field.

### 2.2 Soil sampling and analysis of chemical properties

Soil samples from the maize rhizosphere were collected at the harvesting stage. After the roots were gently shaken to remove the loosely attached soil, the soil that adhered to the roots was brushed off to retain the rhizosphere soil. Three soil subsamples were collected from each pot and thoroughly combined to create a composite sample. Each composite sample was separated into three portions: one portion was air-dried and sieved through a 2-mm mesh for determining soil pH, soil total organic carbon (SOC), and total nitrogen (TN); the second portion was stored at 4°C for microbial biomass carbon (MBC) and microbial biomass nitrogen (MBN), dissolved organic carbon (DOC) and dissolved organic nitrogen (DON), soil available nitrogen (AN), available phosphorus (AP), and available potassium (AK) analysis; the third portion was stored at –80°C for DNA extraction.

The soil pH was assessed with a pH meter (1:5 weight/volume). The SOC content was determined by the potassium dichromate oxidation method. TN was determined by the Kjeldahl method (Bremner and Jenkinson, 1960). MBC and MBN were determined by the chloroform fumigation extraction method (Jenkinson et al., 2004). DOC and DON were analyzed in nonfumigated samples via MBC and MBN analyses. AN was determined by the alkaline hydrolysis diffusion procedure. The AP content was determined by ammonium molybdate spectrophotometry after extraction using the hydrochloric acid-ammonium fluoride method. AK was determined by flame photometry after extraction using the ammonium acetate method (Mulumba and Lal, 2008).

### 2.3 DNA extraction and sequencing

Soil DNA was extracted from 0.5 g of each fresh soil sample using the Quick Soil Isolation Kit (Omega, USA). The quantity and quality of the extracted DNA were measured using NanoDrop 2000 and TBS-380, respectively. The integrity of the extracted DNA was verified via electrophoresis using a 1% agarose gel. The bacterial 16S rRNA gene was amplified using a primer set of 338F (5’-ACTCCTACGGGAGGCAGCAG-3’) and 806R (5’-GGACTACHVGGGTWTCTAAT-3’) (Chen et al., 2016), and the fungal ITS region was amplified using ITS1F (5’-CTTGGTCATTTAGAGGAAGTAA-3’) and ITS2R (5’-GCTGCGTTCTTCATCGATGC-3’) (Yao et al., 2017). The PCR systems and conditions used were the same as those described by Yang et al. (2019). The PCR products were extracted using 2% agarose and purified using the AxyPrep DNA Gel Extraction Kit (Axygen Biosciences; Union City, CA, USA). The purified PCR products from all samples were sequenced using the Illumina MiSeq PE300 platform (Illumina, Inc.). The sequences were processed, and their quality was checked using QIIME2. Sequences that were identified as chimeric or of substandard quality were eliminated through the application of the denoising function (chimera-method consensus) within the DADA2 plugin. Based on the Silva 16S rRNA database (v138) for bacteria and the UNITE (v8) database for fungi, the Ribosomal Database Project (RDP) classifier was used to annotate the species of the representative sequences of the ASV. Alpha-diversity and beta-diversity indices were calculated based on the ASV table. PICRUSt2 and FUNGuild were used to predict the functions of the 16S rRNA and ITS sequences, and the functional information of the bacteria and fungi was obtained. The raw sequences of bacteria and fungi were submitted to the NCBI Sequence Read Archive (SRA) with accession number PRJNA1204484 and PRJNA1209610.

### 2.4 Community assembly process analysis

The bacterial and fungal community assembly processes were investigated by performing null model analyses (Khan et al., 2023). The β-nearest taxon index (βNTI) and Bray-Curtis-based Raup-Crick metric (RC_bray_) were calculated using the “picante” and “ape” R (version 4.4.2) software packages. These computed βNTI and RC_bray_ values facilitated the examination of phylogenetic turnover within microbial communities. A βNTI value exceeding 2 or falling below -2 indicates that deterministic processes are influential in shaping microbial community assembly. βNTI > 2 suggests heterogeneous selection (Hes), whereas βNTI < –2 points to homogeneous selection (Hos). Stochastic processes significantly influence community assembly processes when –2 < βNTI < 2. The RC_bray_ values were subsequently used to quantify the role of specific stochastic processes. When –2 < βNTI < 2 and RC_bray_ > 0.95, the assembly process is dispersal limitation (DL); when –2 < βNTI < 2 and –2 < RC_bray_ < 2, the assembly process is undominated (DR); when –2 < βNTI < 2 and RC_bray_ < –0.95, the assembly process is homogenizing dispersal (HD) (Liu et al., 2020). The neutral community model (NCM) was employed to assess the contribution of neutral processes to the community assembly, which was calculated using the “Hmisc”, “minpack.lm”, and “stats4” packages in R (version 4.4.2).

### 2.5 Statistical analysis

One-way and two-way analysis of variance (ANOVA) and the Duncan multiple range test were performed in SPSS 23.0 to ascertain the statistical significance of differences in soil microbial alpha diversity across treatments (p < 0.05). Beta diversity was assessed by the Bray-Curtis distance and analyzed via nonmetric multidimensional scaling (NMDS). Analysis of similarities (ANOSIM) was performed in R (version 4.4.2) using the package “vegan”. Differences between microbial genera were identified using the linear discriminant analysis (LDA) effect size (LEfSe), with an alpha value > 0.05 and an LDA > 2.8. Co-occurrence networks were constructed using the “igraph”, “Hmisc”, and “qvalue” R (version 4.4.2) software packages. The microbial genera with high relative abundance (total relative abundance > 0.003) and high frequency (observed in more than three samples) were used for network analysis. Pairwise Spearman’s correlations between microbial genera, adjusted *P* values < 0.05, Benjamini-Hochberg false discovery rate (FDR) correction for multiple testing, and correlation coefficients > |0.7| were used to generate the networks. Gephi (version 9.2) was used to calculate the network topological parameters (node, edge, average degree, betweenness centrality, clustering coefficient, average path distance, and modularity) and visualize the co-occurrence network. The associations between soil physiochemical properties and soil microbes were evaluated using the Mantel test using the “ggcor” package. The partial least squares path model (PLS-PM) was used to determine the relationships between microbial groups and management practices. The path coefficients and coefficients of determination (R^2^) derived from this model were validated using SmartPLS (version 4.1.0.0.).

## 3 Results

### 3.1 Soil bacterial and fungal diversity and community composition

**Figure 1:**
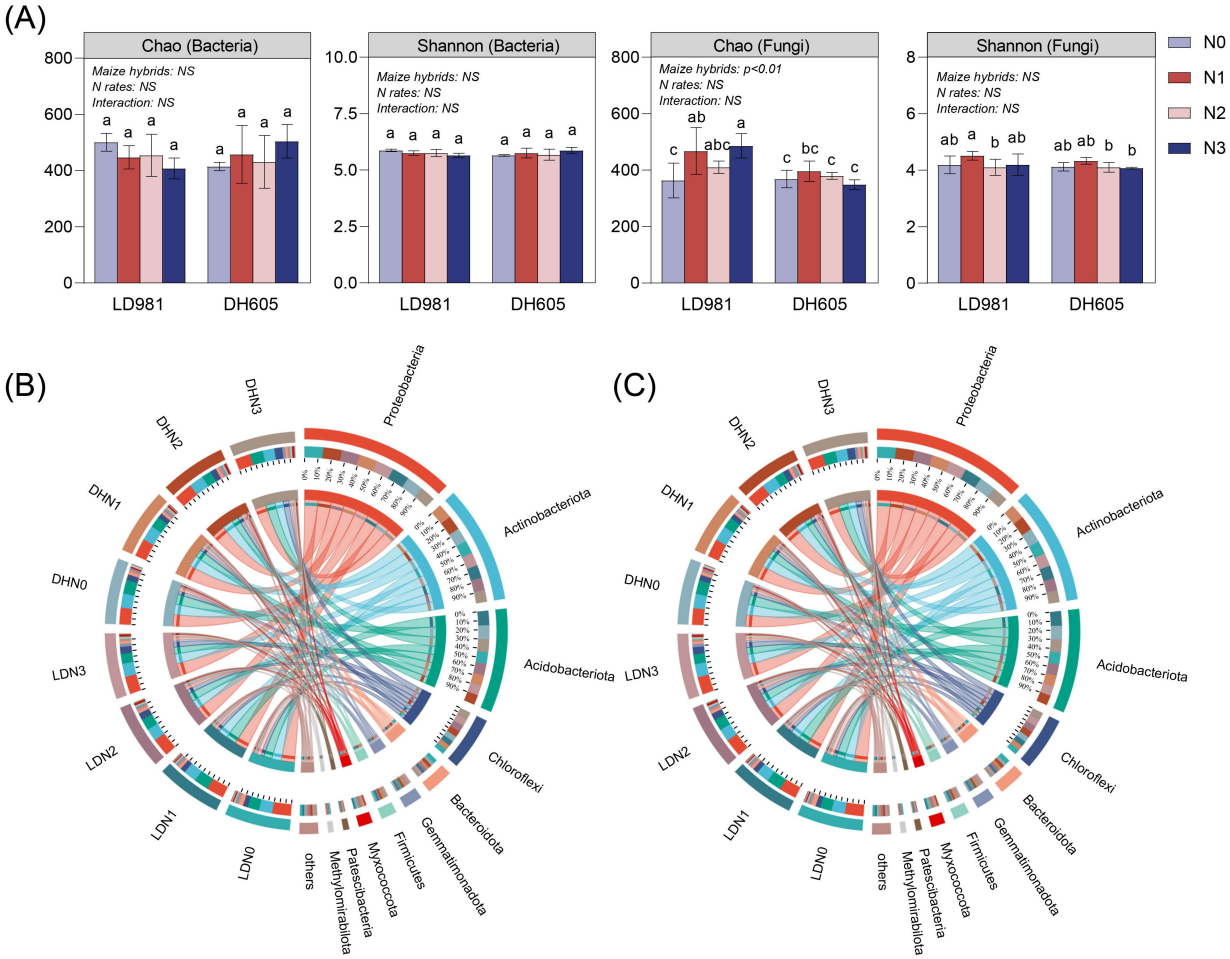
The alpha diversity and community composition of soil bacteria and fungi. **(A)** Chao richness and Shannon’s diversity indices of soil bacteria and fungi. Bars sharing the same lowercase letter indicate no significant difference (p > 0.05). NS represents P > 0.05; * indicates P < 0.05; ** indicates P < 0.01 based on two-way analysis of variance. **(B)** Identification of bacterial phyla through 16S rRNA gene amplicon sequencing. **(C)** Identification of fungal phyla through ITS gene amplicon sequencing.

The influence of maize hybrids on microbial α diversity was significant (**Figure 1A**). Compared to DH605, the variety LD981 led to greater fungal richness (Chao 1). The analysis of bacterial and fungal phylum compositions (**Figures 1B, C**) revealed that maize hybrids did not significantly affect the dominant bacterial or fungal phyla. Compared to those in the N0 and N1 rates, the relative abundance of Chloroflexi in the N2 and N3 rates increased. The relative abundances of Gemmatimonadota and Firmicutes at the N3 level were the highest among the four N rates (**Supplementary Figure S1**). N1 significantly increased the relative abundance of Ascomycota (**Supplementary Figure S1**). To further elucidate microbial community differences across various treatments, LEFSe was employed to identify metagenomic biomarkers (**Supplementary Figure S2**). Specific microbial communities were enriched under different maize hybrids. The dominant genera *Mesorhizobium*, *Candida*, *Metarhizium*, *Arachnomyces*, and *Lectera* were significantly more abundant in LD981 than in DH605, and the genera *Humicola*, *Staphylotrichum*, and *Myxotrichum* were significantly more abundant in DH605 than in LD981. Among the different N rates, N0 and N3 significantly enriched 20 and 24 marker bacterial genera, respectively. Six fungal genera, including *Fusicolla*, *Neonectria*, and *Coniochaeta*, were significantly enriched at the N1 level.

### 3.2 Soil bacterial and fungal assembly

**Figure 2:**
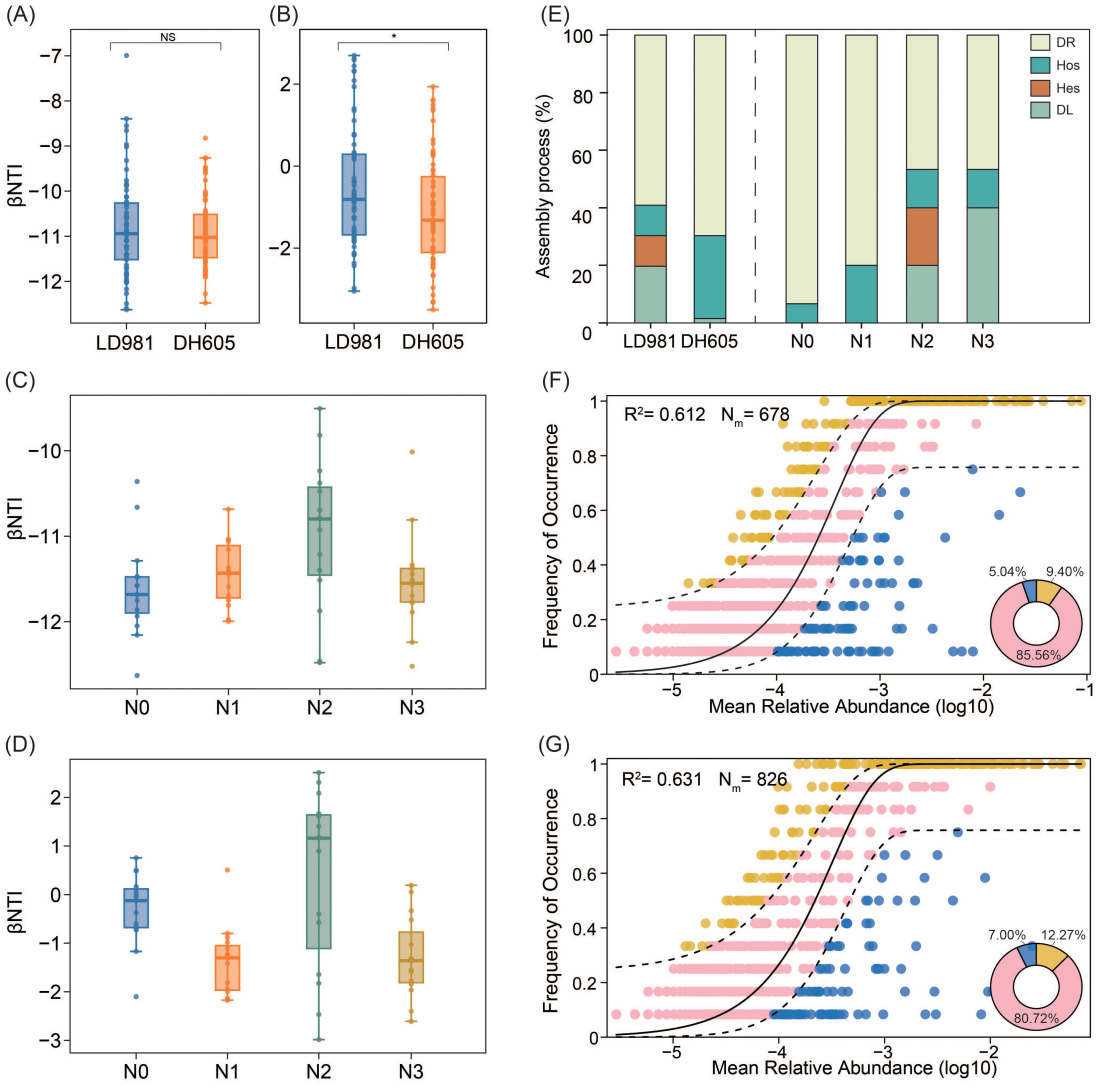
Assembly processes of the patterns of the microbial community. b nearest taxon index (bNTI) in bacteria **(A, C)** and fungi **(B, D)** across the two maize hybrids and four nitrogen rates. NS denotes non-significance (p > 0.05), and * indicates significance (p < 0.05), based on one-way ANOVA results. Contributions of ecological assembly processes dominating fungal **(E)** community turnover. Fungal community assembly process measurements by the NCM of LD981 **(F)** and DH605 **(G)**. ASVs that occurred more frequently than expected by the model are marked in yellow, whereas ASVs that occurred less frequently than predicted by the model are marked in blue. ASVs that occurred within the prediction are shown in pink. The dashed lines represent 95% confidence intervals around the model prediction (black line).

Findings from the null model analyses revealed that deterministic processes, particularly homogeneous selection (βNTI < –2), played a significant role in bacterial community assembly across both the maize hybrid and the four nitrogen rates (**Figures 2A, C**). Stochastic processes (–2 < βNTI < 2) were critical for fungal community assembly in both maize hybrids, especially at LD981 (**Figure 2B**). Stochastic processes accounted for the largest proportion of fungal community assembly at the N0 rate (93.33%), followed by the N3 rate (86.67%) (**Figure 2E**). The proportion of deterministic processes increased as the N1 and N2 rates increased. The homogeneous selection processes accounted for 20.00% of the fungal community assembly at the N1 rate. At the N2 rate, homogeneous selection and heterogeneous selection processes accounted for 13.33% and 20.00%, respectively (**Figure 2E**). The neutral-based model assessment of bacterial community assembly indicated an R^2^ value lower than 0, suggesting a poor fit to the observed data and underscoring that deterministic process predominantly influenced bacterial community assembly over stochastic factors (**Supplementary Table S1**). Based on R^2^ values (0.612 and 0.631 for LD981 and DH605, respectively; 0.534, 0.537, 0.469, and 0.476 for N0, N1, N2, and N3, respectively) and the proportions of outlying taxa beyond the dashed line (14.44% and 19.27% for LD981 and DH605, respectively; 22.99%, 21.94%, 20.06%, and 14.96% for N0, N1, N2, and N3, respectively), the dominant test revealed that the fungal community assemblages of each maize hybrid and N rate were well-described by neutral-based models (**Figures 2F, G**; **Supplementary Figure S3**). The simulation results of the two models were consistent.

### 3.3 Co-occurrence network analysis

**Figure 3:**
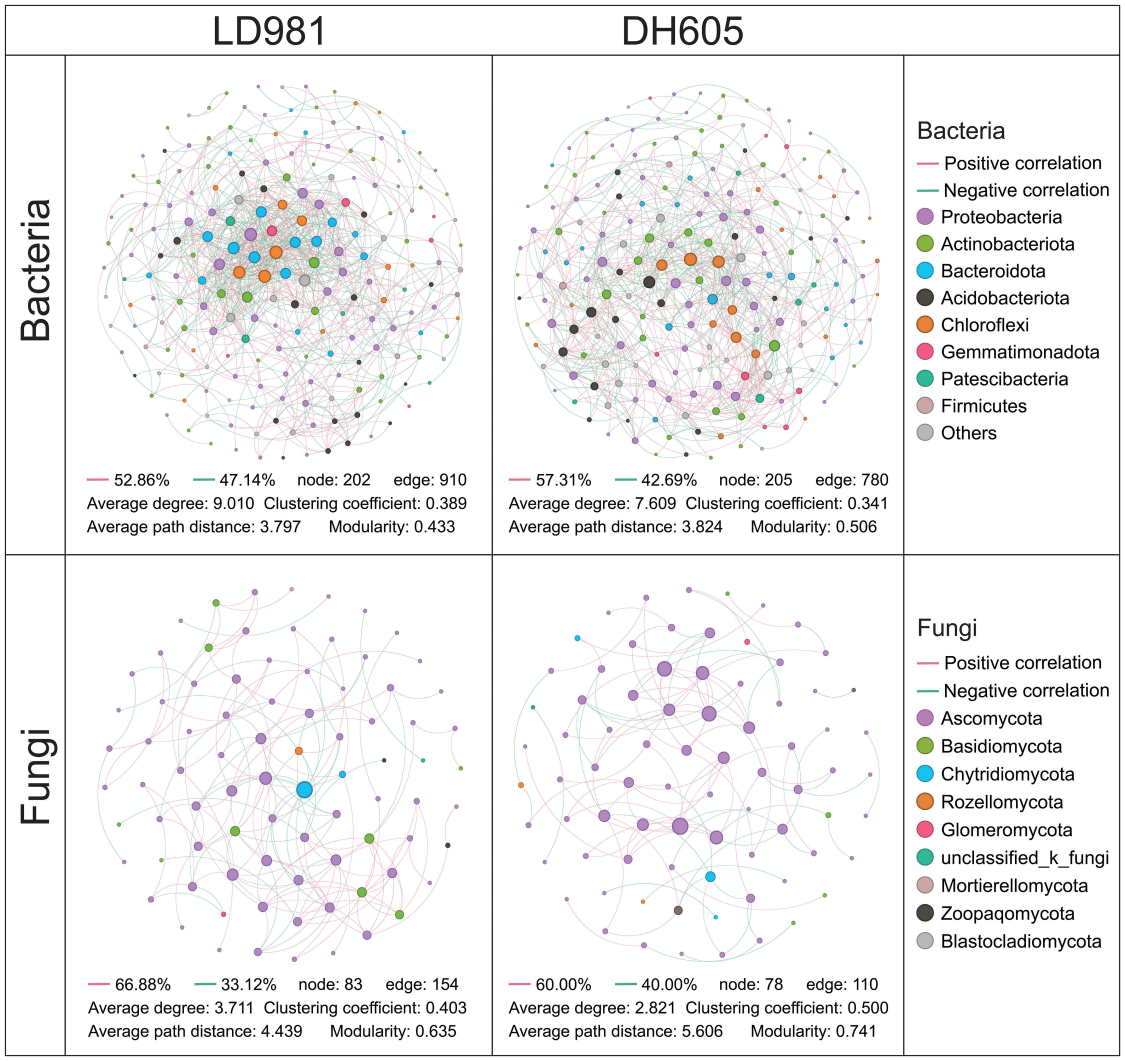
The soil microbial networks associated with the two maize hybrids are displayed at the genus level. Each connection signifies a strong (Spearman’s correlation coefficient ≥0.7) and statistically significant (P < 0.05) correlation. Each node represents a unique genus.

Distinct co-occurrence patterns were found for the soil microbial communities of various maize hybrids (**Figure 3**). In the bacterial network, the average path distance and modularity were greater in DH605 than in LD981. The average degree and clustering coefficient were greater in LD981 than in DH605. In the fungal network, the tendencies of the average path distance, modularity, and average degree of the two maize hybrids were consistent with those in the bacterial network. The clustering coefficient of the fungal network was 24.07% greater in DH605 than in LD981 (**Figure 3**). The co-occurrence network of bacteria and fungi at various N rates also differed (**Supplementary Figure S4**). The lowest values of the clustering coefficient in the bacterial and fungal networks were recorded in N0. N1 had the highest average path distance and modularity and the lowest average degree in the bacterial network, whereas the lowest modularity in the fungal network was observed in N1. The highest values of the clustering coefficient and modularity were found at the N2 level. The highest average degree and lowest average path distance were found in N3 (**Table 1**).

**Table 1:** Parameters of the soil microbial co-occurrence network under various N rates.

	Bacteria				Fungi			
	N0	N1	N2	N3	N0	N1	N2	N3
Positive correlation (%)	54.55	51.81	51.38	53.30	62.80	52.83	61.97	58.07
Negative correlation (%)	45.45	48.19	48.62	46.70	37.20	47.17	38.03	41.93
Node	215	211	218	220	92	97	95	96
Edge	1738	1517	1843	2032	293	318	305	322
Average degree	16.17	14.38	16.91	18.47	6.37	6.56	6.42	6.71
Clustering coefficient	0.48	0.49	0.49	0.48	0.48	0.53	0.56	0.49
Average path distance	2.87	2.95	2.90	2.83	3.57	3.67	3.69	3.55
Modularity	0.54	0.55	0.53	0.46	0.62	0.60	0.67	0.61

### 3.4 Prediction of bacterial and fungal functions

**Figure 4:**
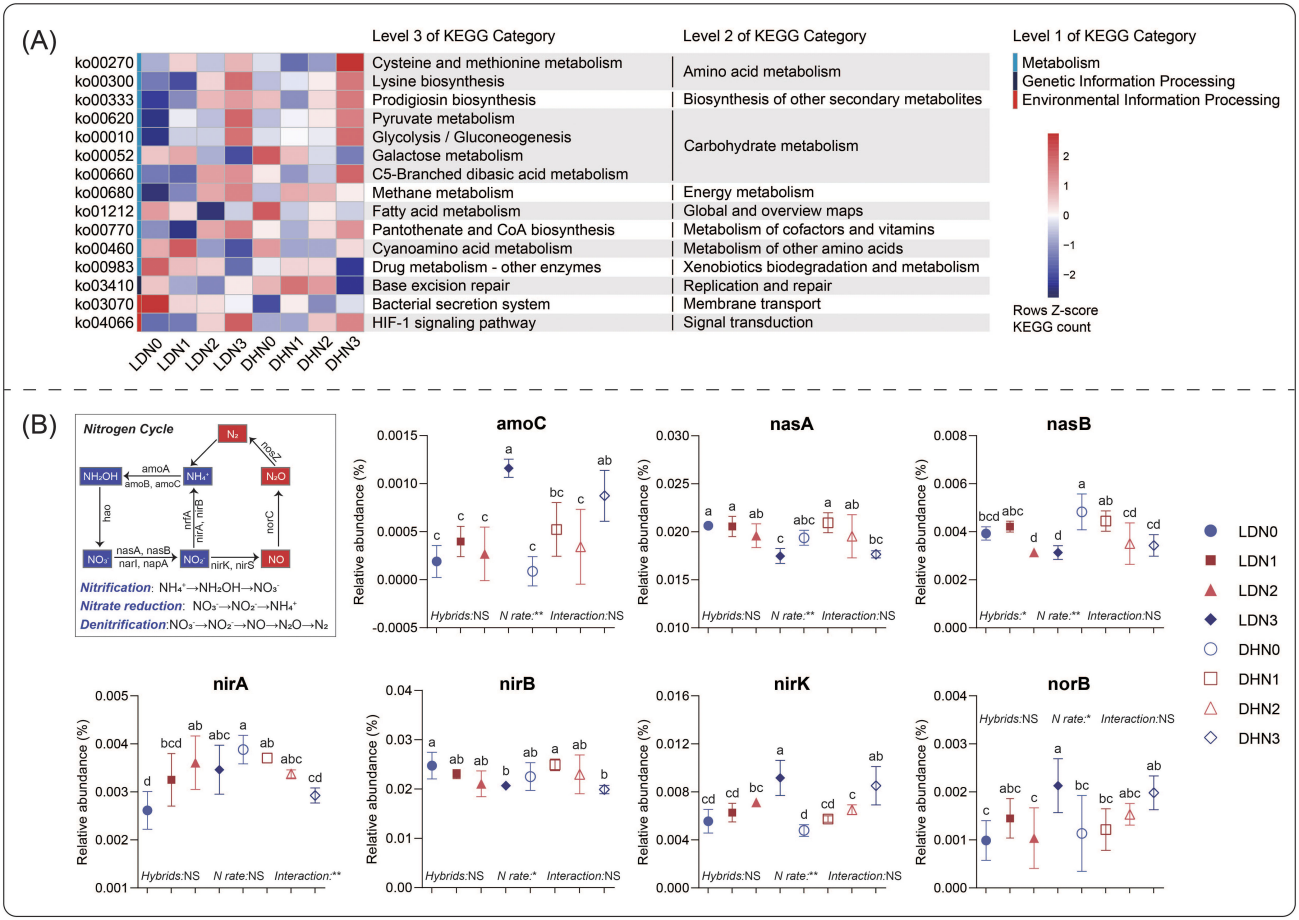
Functional characteristics of the bacterial community based on PICRUSt2. **(A)** Pathways with significant differences between maize hybrids or N rates were determined by ANOVA. **(B)** Differences in functional genes involved in the microbial nitrogen cycle.

The results of PICRUSt2 prediction revealed that the N rates significantly influenced bacterial function (**Figure 4A**). N3 led more sequences to be involved in cysteine and methionine metabolism, pyruvate metabolism, prodigiosin biosynthesis, and lysine biosynthesis than the other N rates. However, fewer sequences involved in galactose metabolism and drug metabolism were found in N3. The functional differences between the maize hybrids were more easily observed at low N rates. DH605 resulted in more sequences involved in cyanoamino acid metabolism than LD981 at the N1 rate. At the N0 rate, more sequences involved in the bacterial secretion system were found in LD981. The microbial genes coding for various N cycles were predicted by PICRUSt2 (**Figure 4B**). The N3 application rate significantly elevated the relative abundances of *amoC*, *nirK*, and *norB* but decreased the relative abundances of *nasA*, *nasB*, and *nirB*. Compared to LD981, DH605 significantly increased the relative abundance of *nasB* at the N0 rate (**Figure 4B**). The fungal functions were predicted by FUNGuild based on the ITS data (**Supplementary Table S2**). The dung saprotrophic-plant saprotrophic fungi at the N0 rate were significantly lower than those at the other N rates. Compared to LD981, DH605 significantly increased the relative abundance of undefined saprotroph-wood saprotroph fungi but decreased the undefined saprotroph fungi (**Supplementary Table S2**).

### 3.5 Identification of important factors influencing changes in microbial groups

**Figure 5:**
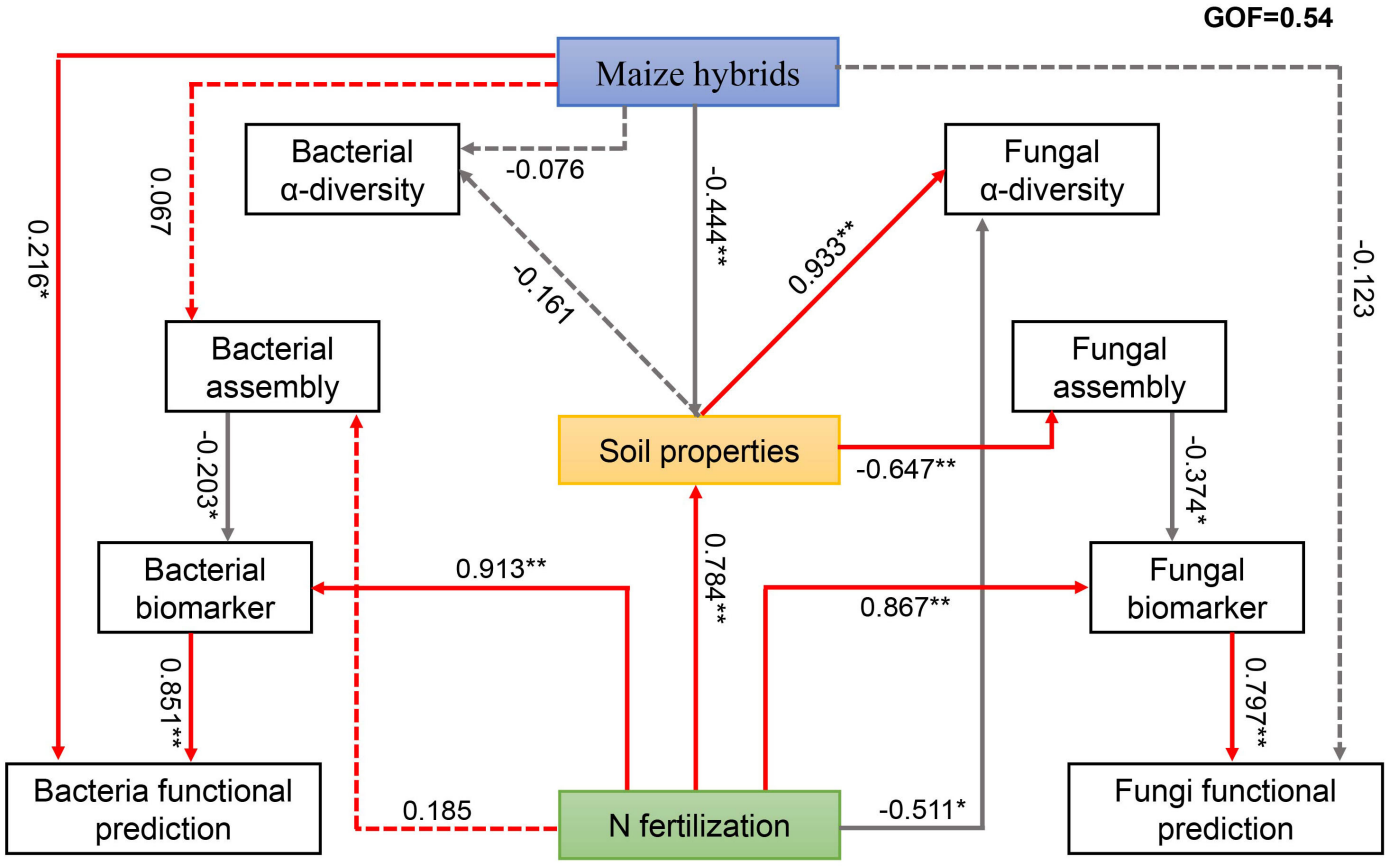
Employs partial least squares path modeling (PLS-PM) to describe the effects of maize hybrid, soil properties, and N fertilization on the soil microbiota. The numbers above/below the arrows indicate the strength of the standardized path coefficient; ** indicates P < 0.01, and * indicates 0.01 ≤ P < 0.05. The solid and dotted lines represent significant and non-significant effects, respectively.

We performed PLS-PM to determine the effects of the maize hybrid and N rate on changes in microbial groups (**Figure 5**). The maize hybrid was significantly correlated with bacterial functional prediction. Through their soil physiochemical properties, maize hybrids affect fungal α diversity and fungal community assembly and ultimately affect the functions of fungi. The N rate was directly positively correlated with bacterial biomarkers and further affected bacterial function. The N rate directly affected fungal α diversity and fungal biomarkers and also affected fungal community assembly through soil physiochemical properties, thereby affecting fungal biomarkers and ultimately influencing fungal functions. The results of the Mantel test revealed strong positive correlations between the pH, DON, and MBN of the LD981 hybrid and the bacterial ASVs. The soil MBC content was strongly correlated with DH605 bacterial ASVs (**Supplementary Figure S5A**). N0 fungal ASVs were strongly correlated with the TN content. N1 fungal ASVs were significantly associated with soil pH. The DON content was strongly correlated with N2 bacterial ASVs, and the TN content was significantly linked to the fungal ASVs at the N2 rate (**Supplementary Figures S5B, D**).

## 4 Discussion

### 4.1 Impacts of maize hybrid and N rates on the soil microbial community

This investigation revealed notable alterations in the composition of bacterial and fungal communities, with N rates exerting a more substantial influence. Sorokin et al. (2014) reported that members of Chloroflexi are involved in the oxidation of NO_2_^–^ during nitrification. The genus *Nitrolancea* in this phylum includes strictly aerobic chemoautotrophic bacteria that use NO_2_^–^ and CO_2_ for growth and can use formate as energy and carbon sources to grow under nutrient-rich conditions (Spieck et al., 2020). The relatively high nitrogen fertilizer at the N2 and N3 rates provided sufficient nutrients, so the relative abundance of Chloroflexi significantly increased compared to that at the low N rate (**Supplementary Figure S1**). High nitrogen application also increased the relative abundances of Gemmatimonadota and Firmicutes (**Supplementary Figure S1**). Gemmatimonadota is associated with intracellular urea hydrolysis and its abundance is positively correlated with the soil nitrogen content (Mujakic et al., 2022). Many Firmicutes strains can act as denitrifiers and produce a positive response when nitrogen application rates are high (Jung et al., 2013).

*Mesorhizobium* is a symbiotic nitrogen-fixing bacterial genus that is significantly enriched in the soil planted with LD981 (**Supplementary Figure S2**). Wang et al. (2019) reported that low-nitrogen conditions are necessary for achieving optimal symbiotic interactions and that LD981 is a representative hybrid with low NUE (Shao et al., 2023). In this study, the significantly enriched marker genus *Sphingomonas* was widely involved in soil nitrogen metabolism at the N0 rate (**Supplementary Figure S2**). *Sphingomonas* sensed the lack of external nitrogen sources at the N0 rate, thereby increasing the expression of nitrogenase and other related genes and increasing the use of external nitrogen sources (Xu et al., 2017). Additionally, the relative shortage of nutrients at the N0 rate also stimulated the stress resistance of maize and significantly enriched the antagonistic bacteria of plant pathogenic fungi such as *Nocardioides* and *Lysobacter* (**Supplementary Figure S2**) (Puopolo et al., 2018; Ribeiro and van der Sand, 2024). *Nitrolancea* and *Nitrosospira*, which were significantly enriched at the N3 rate, were widely involved in the soil nitrogen cycle (**Supplementary Figure S2**). Members of both genera can produce N_2_O through nitrification and denitrification and are sensitive to the nitrogen content in soil (Su et al., 2023). The high nitrogen level at the N3 rate increased the risk of greenhouse gas N_2_O emissions by increasing the relative abundance of *Nitrolancea* and *Nitrosospira* (Deng et al., 2024).

### 4.2 Effects of maize hybrid and N rates on soil microbial community assembly

Understanding microbial assembly processes is necessary to gain deeper insights into ecosystem diversity and function (Zhou and Ning, 2017). The present study revealed that deterministic processes, specifically homogeneous selection, predominantly governed the microbial assembly of the two maize hybrids across the four N rates (**Figure 2**). These findings indicated that the environmental filtration process controls the aggregation of soil rhizosphere bacterial communities. Homogeneous selection arises from similar environments that exert considerable selective pressures, indicating that there was less variation in bacterial community structure than expected to occur by chance in this study (Stegen et al., 2015). This finding was similar to that reported by Yu et al. (2019), who analyzed changes in bacterial community assembly under prolonged fertilization and reported that environmental filtering, rather than stochastic processes, predominantly influences microbial community assembly within agroecosystems. Due to the broader biological niches of bacteria and their rapid growth rates, they are adept at colonizing diverse environments, enabling them to effectively ‘track’ suitable environments dictated by deterministic processes (Chen et al., 2021). Unlike bacteria, fungal communities associated with the two maize hybrids and the four N rates were primarily influenced by stochastic processes (**Figure 2**). Within microbial communities, fungi typically exhibit lower population sizes than bacteria, rendering them more susceptible to random demographic fluctuations and local extinctions (Zhang et al., 2021). The smaller population size and limited dispersal ability of fungi may also increase the effect of stochasticity and drift on fungal community (Larsen et al., 2023). Some studies have shown that communities dominated by stochastic processes may have more species that can use various resources (Li et al., 2023). DL in stochastic processes limits native species and different microbial compositions and also promotes the exploitation of native resources by microorganisms (Cline and Zak, 2014). In this study, the DL process of LD981 was greater than that of DH605 (**Figure 2**). Shao et al. (2023) reported that the ability of LD981 maize to utilize N in soil was lower than that of DH605 maize, which may retain more available N for microorganisms. Among the four N rates, the DL process at the N3 rate with the highest N addition rate accounted for the highest proportion, followed by N2 (**Figure 2**), which may also be due to the greater amount of N nutrients available to microorganisms in the soil.

### 4.3 Impact of maize hybrid and N rate on the soil microbial co-occurrence network

The soils planted with DH605 had greater modularity and a lower average degree of bacterial and fungal networks (**Figure 3**), which was indicative of greater network stabilizing properties (de Vries et al., 2018). High modularity and low connectivity have the potential to confine disturbances to specific modules, thereby inhibiting their propagation throughout the entire network (Xiao et al., 2023). Moreover, the greater average path distance in DH605 (**Figure 3**) implied that the environmental disturbance was transmitted to the whole network more slowly, resulting in better buffering of adverse conditions (Deng et al., 2019). Shao et al. (2023) concluded that DH605 had greater NUE than LD9081 under nitrogen-deficient and excess conditions. This may be the result of a more stable bacterial and fungal network in DH605. The smaller impact of adverse conditions on these microbial networks fosters a more stable and conducive soil micro-environment for maize (Cornell et al., 2023).

Numerous investigations have highlighted the influence of inorganic N fertilizer application as a crucial determinant of the soil microbial co-occurrence network (Ji et al., 2023). Adding excess N may not only jeopardize the stability of the microbial network but also increase the sensitivity of the microbial community (Li et al., 2021). The higher average degree and lower modularity at the N3 rate confirmed this view (**Table 1**). Adding excess nitrogen can change the bacterial and fungal communities by recruiting nitrogen-related microbes and may also increase the abundance of various mutualistic microbes (**Supplementary Figure S2**, **Table 1**). However, as noted in previous research, the presence of negative interactions within microbial networks can enhance overall stability, as competitive dynamics can stabilize co-oscillations within microbial communities (Coyte et al., 2015). The increase in this cooperative relationship is also reflected in the N0 rate (**Table 1**). Without nitrogen application, microorganisms strengthen cooperation with each other by enriching more marker genera to help them survive (Kang et al., 2024). An appropriate nitrogen addition rate of N2 and N3 increased the degree of competition between microorganisms and was more favorable for maintaining the stability of the microbial community network (Zhou et al., 2020). The elevated clustering coefficient and average path length at the N1 and N2 rates also indicated that the microbial network was close and stable (**Table 1**) (Yuan et al., 2023).

### 4.4 Effect of maize hybrid and N rate on the potential function of microorganisms

Cysteine (Cys) and methionine (Met) contain nitrogen and sulfur and are pivotal constituents of low-molecular-weight organic matter in soil (Ma et al., 2022). This organic matter can be swiftly mineralized by soil microorganisms within a few seconds to minutes, although it may take several hours in some cases (Kuzyakov and Xu, 2013). The pathways associated with cysteine and methionine were significantly enhanced at the N3 rate (**Figure 4**), probably because a large amount of exogenous N increased the organic matter degradation by microorganisms (Zhang et al., 2020). Moreover, an increase in cysteine and methionine metabolism can provide nitrogen and sulfur nutrients for plants. The mineralization process provides highly bioavailable inorganic ions that serve as critical nitrogen and sulfur sources for plant growth (Ma et al., 2020). Pyruvate is the final product of glycolysis, which connects glycolysis and the tricarboxylic acid (TCA) cycle. It is a critical intermediate that can be used in the degradation of proteins, fats, and carbohydrates (McCommis and Finck, 2015). Pantothenate and CoA are essential for microbial energy metabolism, cell wall construction, and various biological processes (Dansie et al., 2014). Adequate nitrogen supplementation at the N3 rate increased the energy metabolism of microorganisms, and the increase in genes involved in glycolysis, pyruvate metabolism, and the pantothenate and CoA biosynthesis pathways also confirmed this finding (**Figure 4**).

An increase in the denitrification functional genes *nirK* and *norB* at the N3 rate indicated an increase in the conversion potential from NO_2_^–^ to N_2_O (**Figure 4**). Therefore, excessive nitrogen application increased denitrification functional genes by enriching nitrogen-related bacteria such as *Nitrolancea* and *Nitrosospira* (**Supplementary Figure S2**), which increased the risk of greenhouse gas N_2_O emissions. This result confirmed the conclusions of previous studies that excessive nitrogen application increases soil nitrogen loss (Qu et al., 2014). The influence of the maize hybrid on microbial functionality was more significant at the low nitrogen level (N0) (**Figure 4**). At the N0 rate, the relative abundances of nasB and nirA were greater in DH605 (N-efficient hybrid) than in LD981 (**Figure 4**), indicating that the transformation potential of NO_3_^–^ to NH_4_^+^ increased. Some studies have indicated that the uptake of NH_4_^+^ may represent a vital strategy for maize to secure adequate N (George et al., 2016; Zhang et al., 2019). This explained the conclusion that the nitrogen-efficient hybrid DH605 exhibited superior NUE relative to the nitrogen-inefficient hybrids LD981 under nitrogen-deficient conditions in another study (Shao et al., 2023).

### 4.5 Linking management practices and soil physiochemical properties with microbial traits

Generally, management practices such as maize cultivation and N fertilization play key roles in maintaining soil microbial health and stability by regulating a range of soil physiochemical properties (Yang et al., 2021). In this study, microbial taxa under specific management practices were strongly correlated with various soil nutrients and chemical indicators. Consequently, linking soil physiochemical properties and management practices with microbial traits is necessary. To identify the links described above, a set of models with PLS-SEM that assessed the relationships among the microbial traits, soil physiochemical properties, and management practices were established. The results revealed that maize hybrids and N fertilization affected the community assembly process of fungi through soil physiochemical properties but had no prominent effects on the community assembly of bacteria, which aligns with the findings regarding community assembly patterns (**Figure 2**). The soil physiochemical properties, especially the soil carbon and nitrogen contents, were significantly correlated with the microbial communities (**Supplementary Figure S5**). Maize hybrid and N fertilization affected microbial α diversity through soil physiochemical properties. However, the effects of management practices on microbial functionality may not be mediated by microbial α-diversity (**Figure 5**). In many studies, microbial α diversity was used as an indicator of soil health, and some researchers suggest that the primary advantage of increased microbial α-diversity lies in functional redundancy (Mendes et al., 2015; Venter et al., 2016; Chou et al., 2024). Microbial α diversity can predict microbial functional diversity, but this does not mean that an absolute relationship is present between the two. The judgment of whether the microbial flora is beneficial should also be combined with its community structure and function (Nannipieri et al., 2020; Zhang et al., 2024a). N fertilization affects microbial functions through specific biomarker genera and functional genes. External N is decomposed and transformed by microorganisms after it is applied to the soil. The recruitment of specific functional microorganisms accelerates the utilization of excess N in the soil and stimulates the degradation of organic matter in the soil (Han et al., 2023; Yu et al., 2023). Management practices, including maize hybrid and N fertilization, significantly influenced microbial community dynamics, which are directly or indirectly reflected in the functions of soil microbes.

## 5 Conclusions

In this study, we systematically summarized the alterations in rhizosphere soil microbial traits associated with different maize hybrids and N rates. Our findings indicated that N rates exert a more pronounced impact on the composition of bacterial and fungal communities compared to maize hybrids, primarily by affecting the relative abundances of Chloroflexi, Gemmatimonadota, Firmicutes, and Ascomycota. The N-efficient hybrid DH605 had a more stable microbial network in contrast to the N-inefficient hybrid LD981. The bacterial community was dominated by deterministic processes (homogeneous selection), whereas stochastic processes played a significant role in fungal community assembly. The dispersal limitation of fungi peaked at the N3 rate, followed by the N2 rate. Under N- excess conditions (N3), the bacterial and fungal community networks were the most complex but unstable, followed by N2, N0, and N1 rates. An excessive nitrogen rate (N3) increased the relative abundance of the denitrification genes *nirK* and *norB* by enriching nitrogen-related genera such as *Nitrolancea* and *Nitrosospira*, and the relative abundance of pathways such as cysteine and methionine metabolism and pyruvate metabolism were significantly increased. The effects of management practices (i.e., maize hybrids and N rates) on microbial communities are directly or indirectly reflected in microbial functions, although this process might not always be mediated by microbial α diversity. The findings of this study underscore the necessity of optimizing N rates through careful adjustments in topdressing, aiming to enhance microbial network stability and mitigate environmental repercussions. The optimum N rate applied in this study was the N2 rate, which improved the rhizosphere soil microbial community and network. Considering the perfect control of pot experimental conditions, future research should consider the long-term effects of climate change on the N utilization characteristics across different maize hybrids at the field scale.

## Data Availability

The datasets presented in this study can be found in online repositories. The names of the repository/repositories and accession number(s) can be found in the article/**Supplementary Material**.
