# Supplementary material for: Rhizosphere-associated bacterial and fungal communities of two maize hybrids under increased nitrogen fertilization
Source: Front Plant Sci. 2025 Mar 3;16:1549995. doi: 10.3389/fpls.2025.1549995 (PMC11911359; doi:10.3389/fpls.2025.1549995)
Supplement: Supplementary file 1 [file DataSheet1.docx]

Supplementary Material


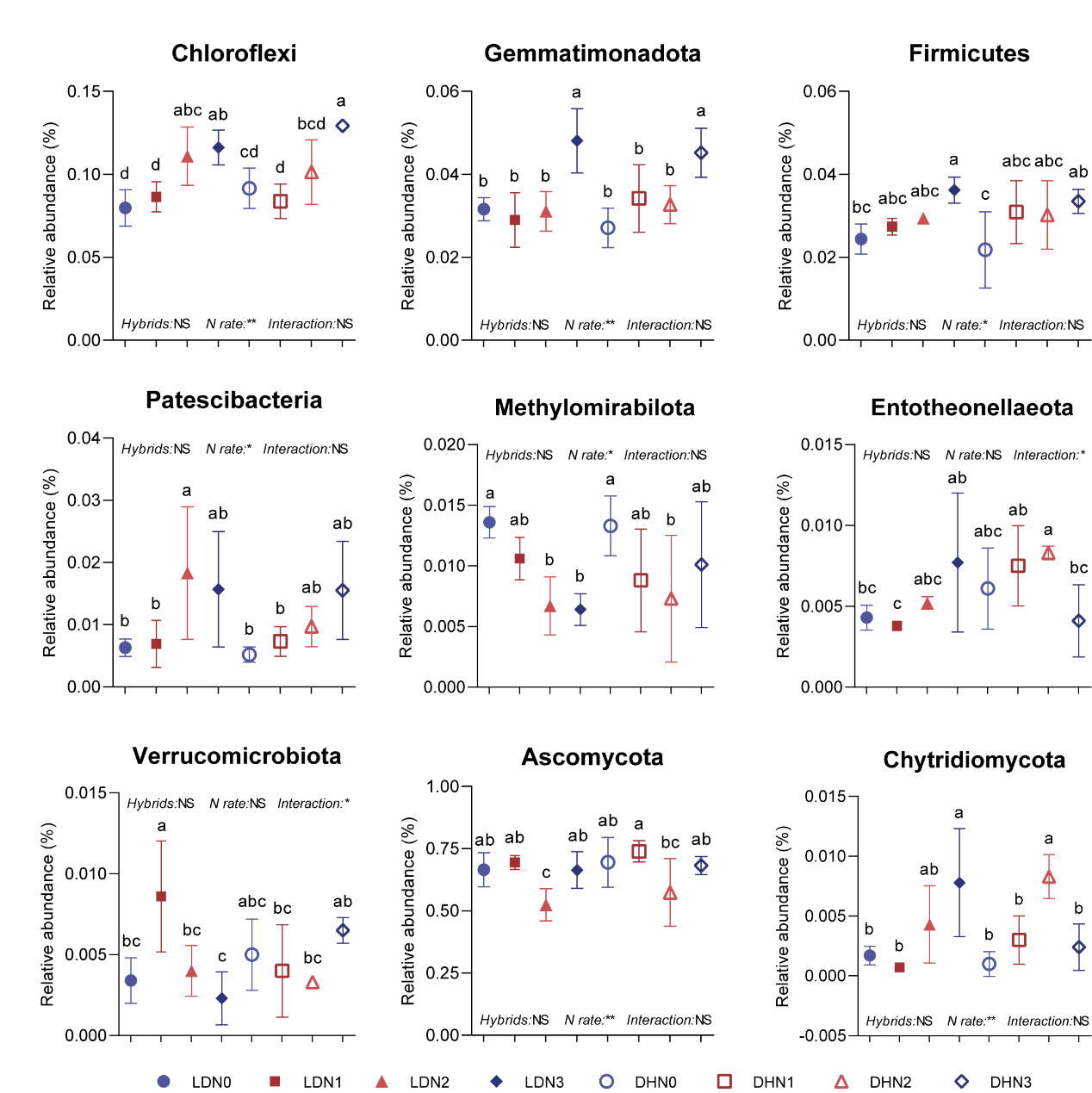


**Supplementary Figure 1.** Bacterial and fungal phylum with significant differences between groups based on ANOVA. Values with the same lowercase letter are not significantly different (p > 0.05). NS was P>0.05; * was P<0.05; ** was P<0.01 based on two-factor analysis of variance.


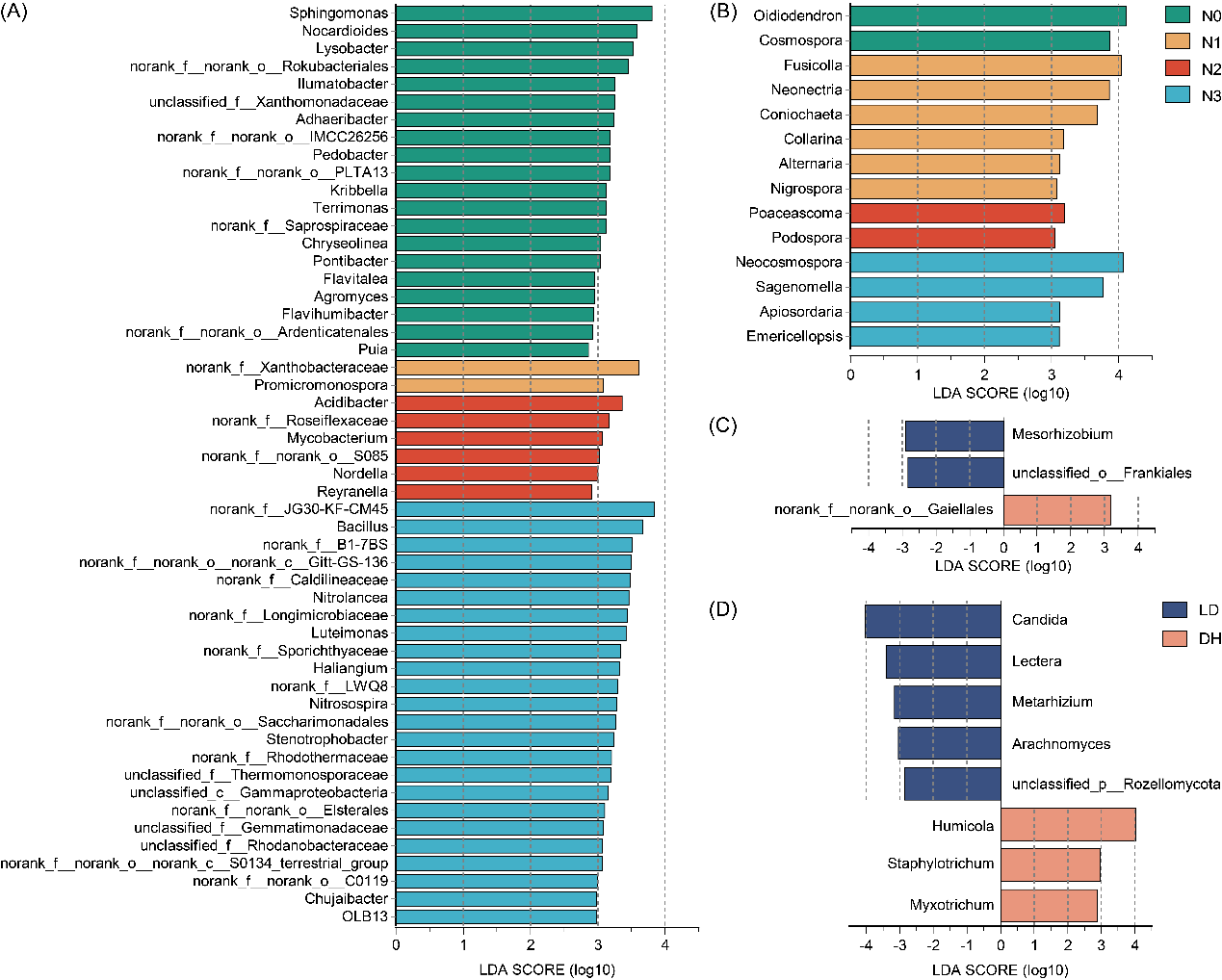


**Supplementary Figure 2.** Significant differences in relative abundances at the genus level as determined by linear discrimination analysis (LDA) effect size (LEFSe). (A) The dominant bacterial genera in different N rates. (B) The dominant fungal genera in different N rates. (C) The dominant bacterial genera in different maize hybrids. (D) The dominant fungal genera in different maize hybrids.


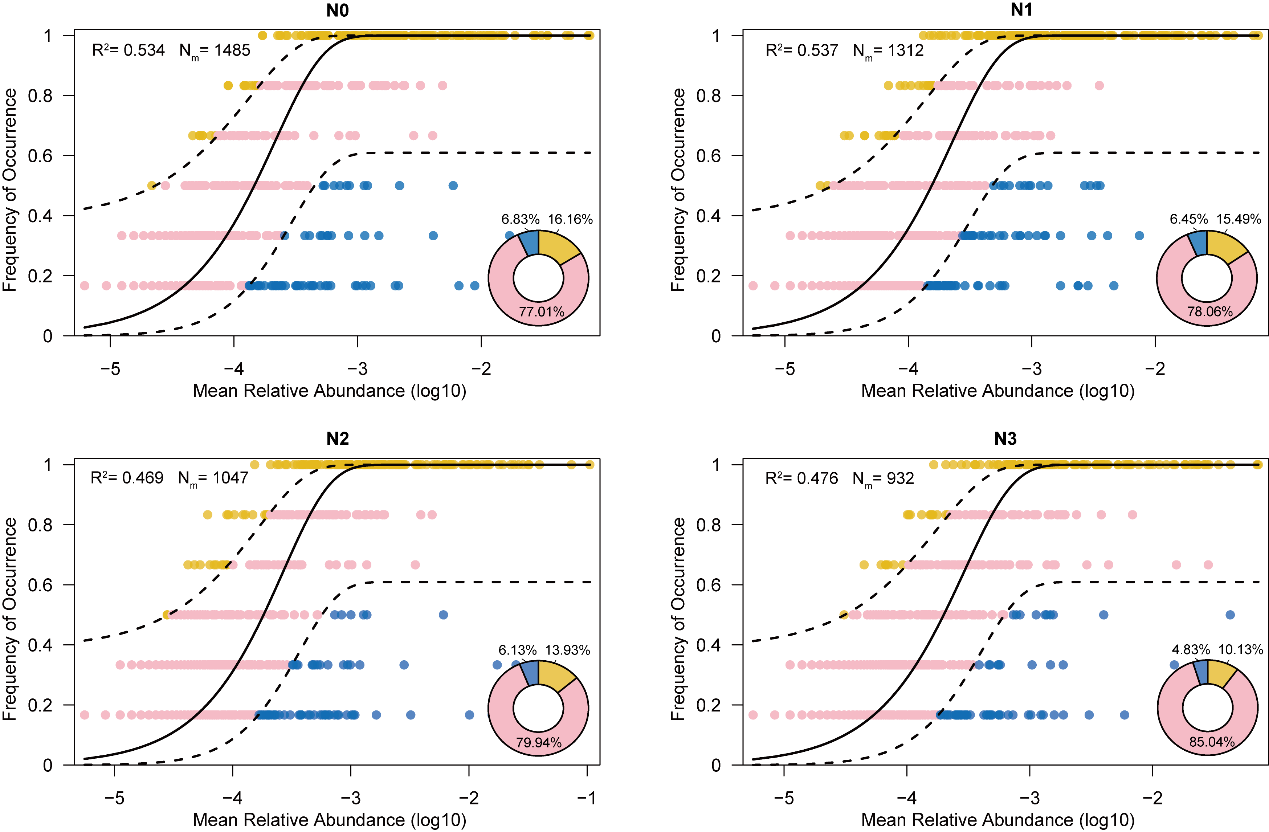


**Supplementary Figure 3.** Fungi community assembly process measurements by the neutral community model (NCM) at different nitrogen rates. ASVs that occur more frequently than predicted by the model are shown in yellow, while those that occur less frequently than predicted are shown in blue. ASVs that occur within prediction are shown in pink. Dashed lines represent 95% confidence intervals around the model prediction (black line).


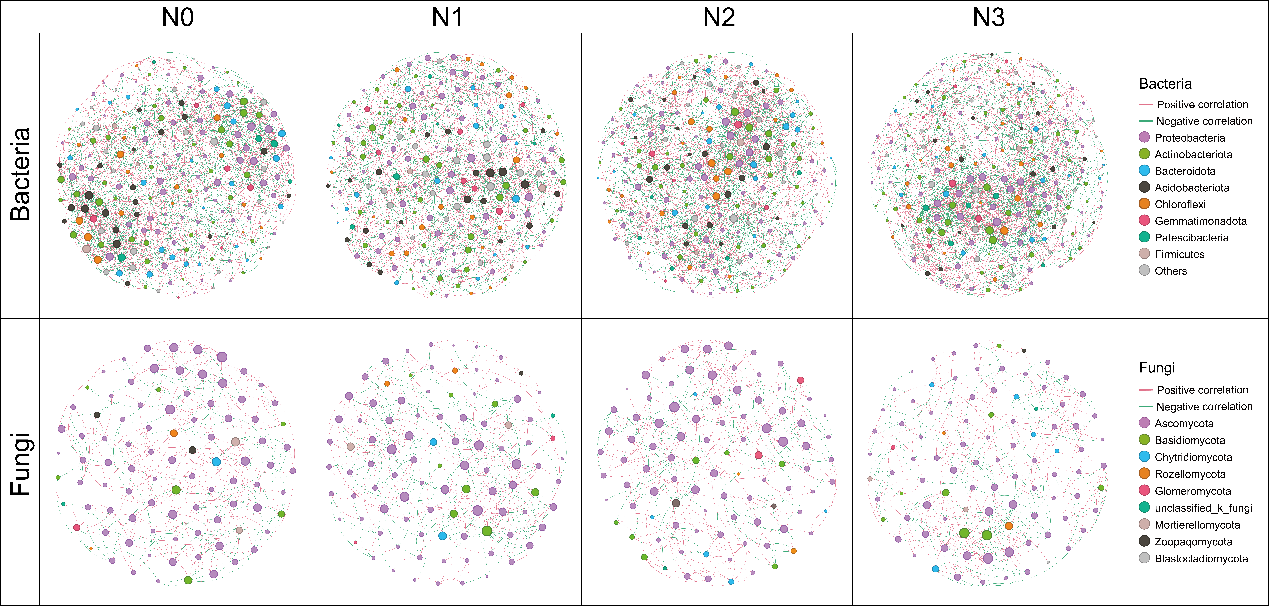


**Supplementary Figure 4.** Soil microbial networks are displayed at the genus level under different N rates. A connection indicates a strong (Spearman’s≥0.7) and significant (P<0.05) correlation. Each node represents a unique genus.


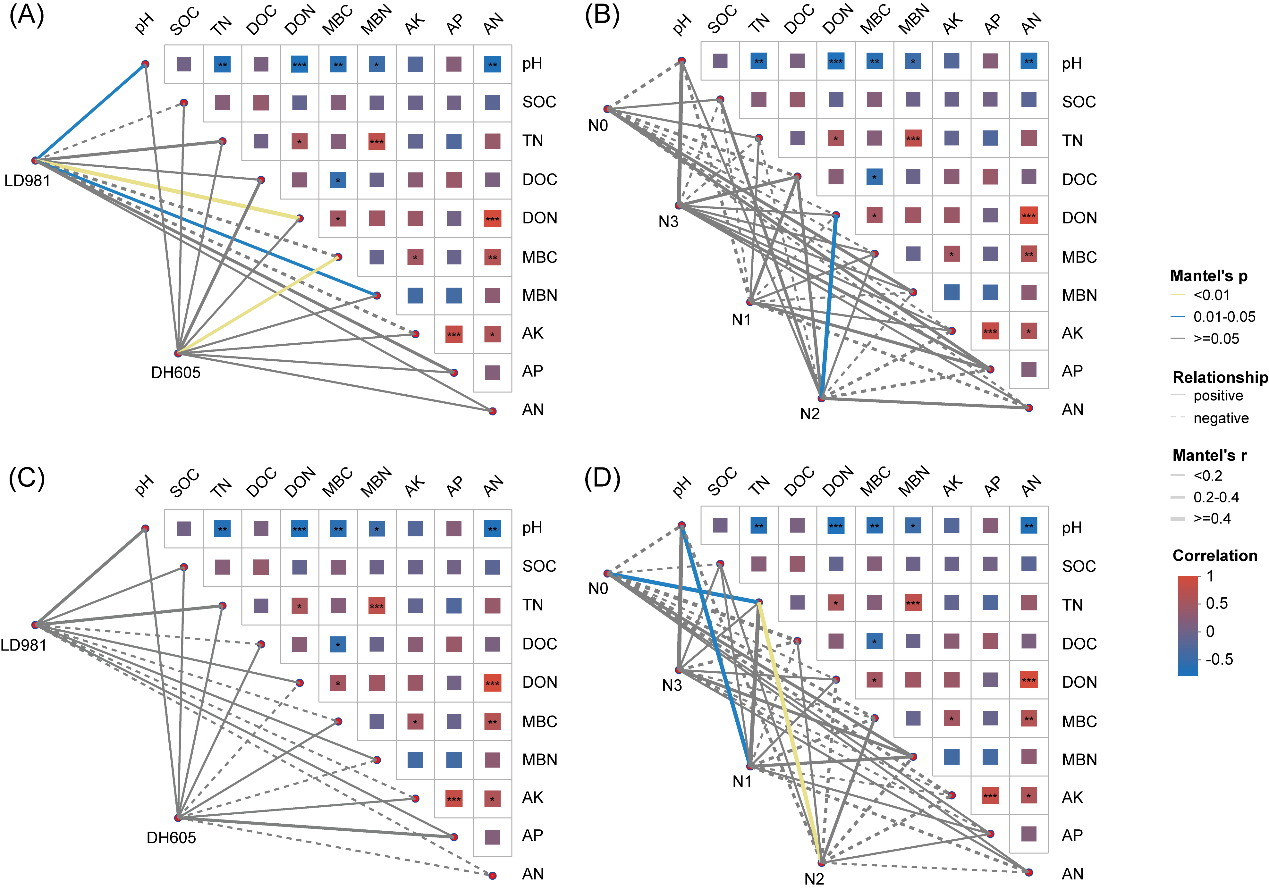


**Supplementary Figure 5.** The relationship between soil microbes and soil properties based on Mantel tests. (A, B) Bacteria of various maize cultivars and N rates are related to soil properties. (C, D) Fungi of various maize cultivars and N rates are related to soil properties.

**Supplementary Table 1.** Fungi community assembly measurements by the normalized stochasticity ratio (NST) ratio index at different treatments.

|  | Treatment | R^2^ | N_m_ |
| --- | --- | --- | --- |
| Maize hybrids | LD981 | -0.625 | 56 |
|  | DH605 | -0.493 | 56 |
| Nitrogen rates | N0 | -1.899 | 64 |
|  | N1 | -1.546 | 66 |
|  | N2 | -2.837 | 60 |
|  | N3 | -1.764 | 65 |

**Supplementary Table 2.** The relative abundance (%) of fungal function based on FUNGuild.

|  | Undefined Saprotroph | Undefined Saprotroph-Wood Saprotroph | Dung Saprotroph-Plant Saprotroph | Dung Saprotroph-Undefined Saprotroph |
| --- | --- | --- | --- | --- |
| LDN0 | 25.97 b | 2.73 b | 0.30 b | 0.33 b |
| LDN1 | 26.27 b | 3.49 ab | 0.81 a | 0.32 b |
| LDN2 | 19.24 c | 2.74 b | 0.71 a | 0.55 b |
| LDN3 | 35.20 a | 2.76 ab | 0.90 a | 0.44 b |
| DHN0 | 22.28 bc | 7.87 ab | 0.52 ab | 0.33 b |
| DHN1 | 25.04 b | 4.41 a | 0.51 ab | 0.41 b |
| DHN2 | 21.98 bc | 3.99 ab | 0.67 a | 0.35 b |
| DHN3 | 23.24 bc | 4.35ab | 0.75 a | 0.82 a |
| Maize cultivars (*A*) | * | * | NS | NS |
| N rates (B) | ** | NS | * | * |
| Interaction (*A×B*) | ** | NS | NS | * |

Note: Values in the same row followed by different lowercase letters are significantly different (p < 0.05, LSD test). * refers to P < 0.05, ** refers to P < 0.01, and NS refers to P > 0.05 based on two-way ANOVA.
